# Theory and Experimental Validation of a Spatio-temporal Model of Chemotherapy Transport to Enhance Tumor Cell Kill

**DOI:** 10.1371/journal.pcbi.1004969

**Published:** 2016-06-10

**Authors:** Zhihui Wang, Romica Kerketta, Yao-Li Chuang, Prashant Dogra, Joseph D. Butner, Terisse A. Brocato, Armin Day, Rong Xu, Haifa Shen, Eman Simbawa, A. S. AL-Fhaid, S. R. Mahmoud, Steven A. Curley, Mauro Ferrari, Eugene J. Koay, Vittorio Cristini

**Affiliations:** 1 Department of NanoMedicine and Biomedical Engineering, University of Texas Medical School at Houston, Houston, Texas, United States of America; 2 Brown Foundation Institute of Molecular Medicine, University of Texas Medical School at Houston, Houston, Texas, United States of America; 3 Department of Imaging Physics, University of Texas MD Anderson Cancer Center, Houston, Texas, United States of America; 4 Department of Pathology, University of New Mexico, Albuquerque, New Mexico, United States of America; 5 Department of Mathematics, California State University, Northridge, California, United States of America; 6 Department of Chemical and Biological Engineering and Center for Biomedical Engineering, University of New Mexico, Albuquerque, New Mexico, United States of America; 7 Department of Nanomedicine, Methodist Hospital Research Institute, Houston, Texas, United States of America; 8 Department of Mathematics, Faculty of Science, King Abdulaziz University, Jeddah, Saudi Arabia; 9 Michael E. DeBakey Department of Surgery, Baylor College of Medicine, Houston, Texas, United States of America; 10 Department of Radiation Oncology, University of Texas MD Anderson Cancer Center, Houston, Texas, United States of America; Università degli Studi di Napoli Federico II, ITALY

## Abstract

It has been hypothesized that continuously releasing drug molecules into the tumor over an extended period of time may significantly improve the chemotherapeutic efficacy by overcoming physical transport limitations of conventional bolus drug treatment. In this paper, we present a generalized space- and time-dependent mathematical model of drug transport and drug-cell interactions to quantitatively formulate this hypothesis. Model parameters describe: perfusion and tissue architecture (blood volume fraction and blood vessel radius); diffusion penetration distance of drug (i.e., a function of tissue compactness and drug uptake rates by tumor cells); and cell death rates (as function of history of drug uptake). We performed preliminary testing and validation of the mathematical model using *in vivo* experiments with different drug delivery methods on a breast cancer mouse model. Experimental data demonstrated a 3-fold increase in response using nano-vectored drug *vs*. free drug delivery, in excellent quantitative agreement with the model predictions. Our model results implicate that therapeutically targeting blood volume fraction, e.g., through vascular normalization, would achieve a better outcome due to enhanced drug delivery.

**Author Summary:**

Cancer treatment efficacy can be significantly enhanced through the elution of drug from nano-carriers that can temporarily stay in the tumor vasculature. Here we present a relatively simple yet powerful mathematical model that accounts for both spatial and temporal heterogeneities of drug dosing to help explain, examine, and prove this concept. We find that the delivery of systemic chemotherapy through a certain form of nano-carriers would have enhanced tumor kill by a factor of 2 to 4 over the standard therapy that the patients actually received. We also find that targeting blood volume fraction (a parameter of the model) through vascular normalization can achieve more effective drug delivery and tumor kill. More importantly, this model only requires a limited number of parameters which can all be readily assessed from standard clinical diagnostic measurements (e.g., histopathology and CT). This addresses an important challenge in current translational research and justifies further development of the model towards clinical translation.

## Introduction

The biological drivers of cancer promote a physical microenvironment that differs significantly from normal tissues [1]. The underpinnings of the dysregulated physical properties of cancer are an active area of investigation. Research efforts in this area are highlighted by modern oncologic concepts of how the physical microenvironment influences tumor behavior (i.e., theories such as Transport Oncophysics and vascular normalization) [2, 3], and are supported by experimental data in animal cancer models and cancer patients [4, 5]. Mathematical modeling has provided quantitative insights into the understanding of how these physical properties impact therapeutic drug transport, which will eventually help to optimize, predict and provide treatment strategy on an individual patient basis [6–10]. Notable modeling successes with validation against *in vitro*, *in vivo*, and/or patient data include mathematical modeling of cancer tumor development [11–14] and traditional drug delivery in neoadjuvant [15, 16], adaptive [17], and standard chemotherapy treatment conditions [18]. We have also studied how mathematical modeling of the mass transport of drugs can mechanistically describe the therapeutic response to chemotherapy [19–22] and enable an understanding of the drug delivery process in humans [23]. However, to date, these efforts have been limited by the inability to account for spatial and temporal heterogeneity in drug dosing and tumor characteristics.

Chemotherapy drugs need to traverse the vasculature, interstitial space (i.e., stroma and microenvironment), and cancer cell membranes to finally reach intracellular targets. At multiple biological scales, tumors have properties that impede the delivery of chemotherapy, and the poor delivery of drugs prevents the killing of cancer cells [3, 24, 25]. In solid tumors, the disorganized and leaky vasculature of the tumor microenvironment influences the delivery of and response to systemic chemotherapy [2, 26–28]. Furthermore, the stroma not only acts as a physical barrier to drug delivery, but also impairs the function of blood vessels [29, 30]. Conceivably, the ability to characterize these physical properties of tumors would enable scientists and clinicians to not only predict response, but also rationally design therapeutics for an individual cancer patient.

Toward this goal, we have developed a theory of chemotherapy response based on the quantitative physical transport properties of cancer cells [20, 21] as well as solid tumors [19, 22, 23]. Our approach describes how a tumor’s biophysical properties affect drug delivery using “quantitative pathology,” a translational approach combining mathematical modeling and measurements from histopathology of individual patients’ tumors to predict treatment outcome [19–22, 31]. In particular, the mechanistic model presented in [19] accurately predicted the fraction of tumor killed (denoted by *f*_kill_) from chemotherapy in patients with colorectal cancer (CRC) metastatic to liver and in patients with glioblastoma. We also demonstrated the feasibility of the model for clinical translation by predicting patient-specific treatment efficacy using computed tomography (CT) scan data [19, 23]. The model presented in [20] is a time-dependent model based on first principles of cell biophysics to predict *in vitro* the nonlinear dose response curves for two types of delivery methods, free drug and targeted nano-carriers. We found that drug delivery mediated by nano-carriers targeting the tumor cells overcomes resistance to free drug because of improved cellular drug uptake rates.

We previously hypothesized that tumor kill would be significantly enhanced through the elution of drug from a nano-carrier that deposits in the tumor [3]. A series of pilot studies have tested this hypothesis using experimental tumor models for delivering drugs via multistage vectors (MSVs) [32–34]; MSVs are nested particles (with smaller particles nested inside larger ones), particularly designed to lodge in tumor vasculature to release nanoparticles over an extend period of time (e.g., several weeks). To date, mathematical modeling validated against experimental laboratory results has resulted in insights into fine-tuning nano-carriers for optimized drug delivery through charge and size optimization [35, 36], drug release duration [37, 38], and targeting molecule surface loading [39]. Work to this end has also resulted in increased understanding of how vasculature structure and the resulting interstitial fluid behavior are involved in distribution of the nano-carriers in the blood vessels *in vivo* [40]. Here, we develop a mathematical model to quantitatively formulate the hypothesis of drug delivery via loaded nano-carriers. Specifically, we extend our previous time-dependent modeling work [20] by also accounting for spatial dependence in predicting tumor response to systemic agents. This generalized model allows us to consider a variety of treatment strategies, including systemic drug delivery via nano-carriers, and helps to predict the tumor response to different forms of drug delivery methods before the start of treatment. Model predictions are further validated using experiments on a breast cancer mouse model *in vivo*.

## Methods

### Generalized mathematical model

**Fig 1:**
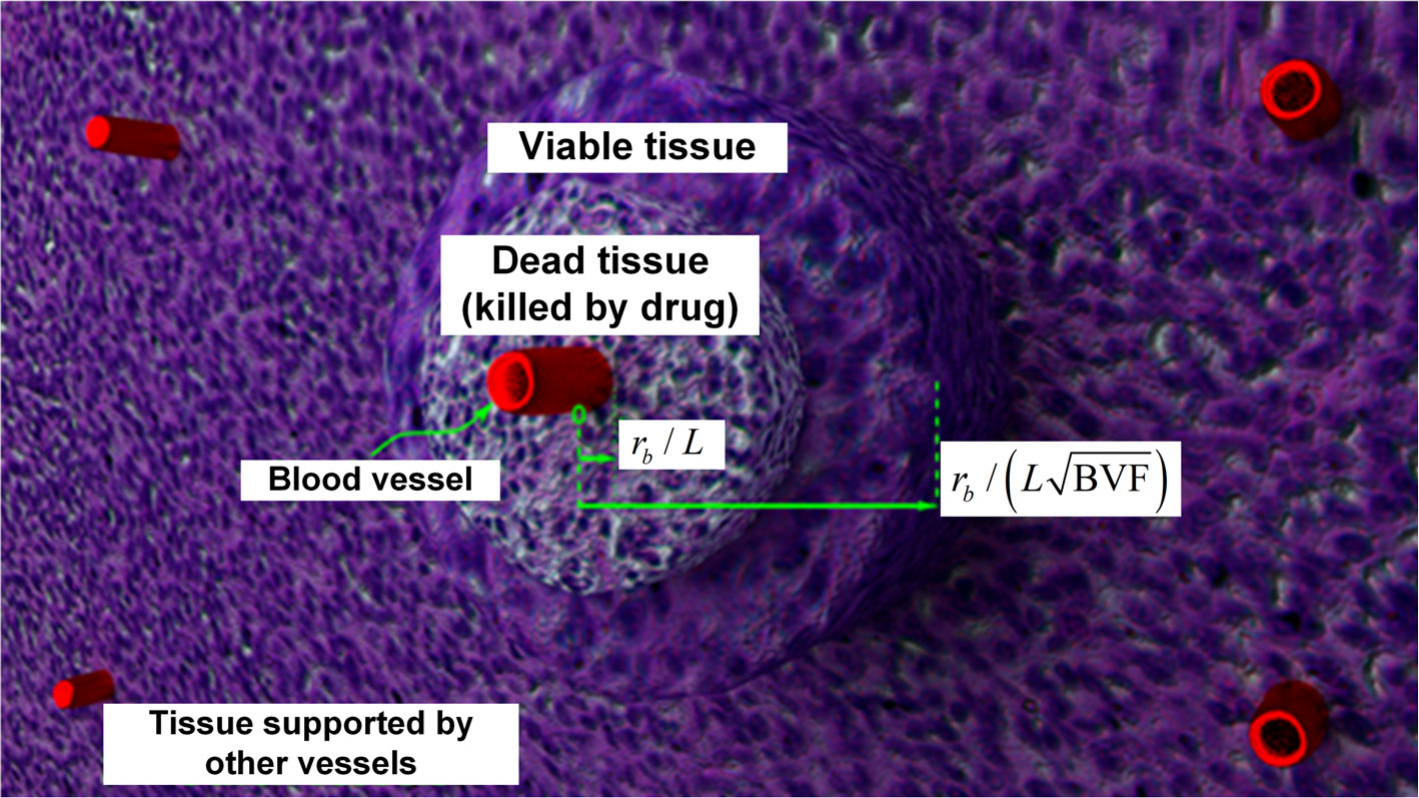
Illustration of transport-based hypothesis. By diffusion, a blood vessel supplies substrates to the cylindrical tissue volume surrounding the vessel. We hypothesize that at each position inside the tissue, the substrate supply is supported by the closest blood vessel. Thus, the influenced tissue surrounding a vessel can be estimated to be between a cylinder of radius *r*_b_ / (*L* BVF^1/2^) in dimensionless form, and the vessel itself with dimensionless radius *r*_b_ / *L*. Theoretically, chemotherapeutic drugs delivered by a blood vessel kill the tissues immediately adjacent to the vessel, leaving some viable tissues on the far end. Here, we propose that through drug-loaded nano-carriers that can accumulate within tumors and continuously release drugs for a longer time (e.g., lasting several cell cycles), the drugs can penetrate further into the surrounding tissue volume and thus achieve a higher tumor killing ratio.

We extend the time-dependent drug-cell interaction model [20] by incorporating spatial dependence to describe perfusion and diffusion heterogeneities. The governing equations for drug concentration *σ*(**x**, *t*) and the volume fraction of tumor cells *φ*(**x**, *t*) are

$$\frac{\partial \sigma }{\partial t}=D\nabla^{2}\sigma -\lambda_{\text{u}}\varphi \sigma ,$$ (1)

$$\frac{\partial \varphi }{\partial t}=-\lambda_{\text{u}}\lambda_{\text{k}}\varphi \left(x,t\right)\int_{0}^{t}\sigma \left(x,\tau \right)\varphi \left(x,\tau \right)\text{d}\tau ,$$ (2)

where *D* is the diffusivity of the drug, *λ*_u_ the per-volume cellular uptake rate of drug, and *λ*_k_ the death rate of tumor cells per unit cumulative drug concentration. While drug uptake is known to have a nonlinear dependency on drug concentration [41, 42], we assume a linear drug uptake for simplicity. Because drug diffusion time and the plasma half-life of drug are both much shorter than the time scale for cell death (on the order of minutes vs. hours or days), and also because the model will be examined on time scales of days to weeks, rather than minutes, Eq 1 can be solved at the steady state (i.e., ∂*σ* / ∂*t* ≅ 0; note that this is actually a quasi-steady state, meaning that *σ*(**x**, *t*) quickly relaxes to the instant steady state defined by *φ*(**x**, *t*)). Thus, without the time derivative in Eq 1, the solution *σ*(**x**, *t*) is independent of initial conditions; for boundary conditions, we set a drug concentration *σ*_0_ at the blood vessel wall. We further clarify that the steady state solution of *σ*(**x**, *t*) still has spatial dependence, and hence a gradient; it is only steady state with respect to time. The main assumption underlying the use of a steady-state equation with a constant boundary condition was that the entire time curve of several bolus injections over several months of treatment for each patient could be satisfactorily replaced with a constant *σ*_0_ equal to the time averaged drug concentration throughout the entire multi-month treatment regimen. That is, we assume that a drug administered as bolus at a certain dose level has the same effect as the same total amount of drug administered over several months at a constant, smaller dose level; see [19, 23] for validation of this assumption (with patient data) on the use of a constant boundary condition. For a cylindrically symmetric domain surrounding a blood vessel (**Fig 1**), the boundary conditions can be set to

$$\sigma \left(r=r_{b},t\right)=\sigma_{0}\;,\;\text{and}$$ (3)

$$n\cdot {\nabla \sigma |}_{x\to \infty }\to 0,$$ (4)

where *r* denotes the radial position from the center of the cylinder, and *r*_b_ represents the blood vessel radius; the second boundary condition reflects that the far-field drug concentration flattens out. Furthermore, Eq 2 has no spatial derivatives, and thus only requires the initial conditions for *φ*(**x**, *t*), which we set to

$$\varphi \left(x,t=0\right)=\varphi_{0},$$ (5)

i.e., a homogeneous initial tumor volume fraction. As detailed below, this generalized model allows us to examine not only successive (conventional) bolus chemotherapy, characterized by a time-varying intravenous drug concentration *σ*_0_ according to a specific dosing and timing regimen, but also drug release through loaded nano-carriers where drugs are released at a nearly constant rate over a certain time interval, approximated here by a constant *σ*_0_.

### Model normalization

The generalized model (Eqs 1 and 2) is nondimensionalized as

$$\nabla^{'}{}^{2}\sigma^{'}-\varphi^{'}\sigma^{'}=0,$$ (6)

$$\frac{\partial \varphi^{'}}{\partial t^{'}}=-\varphi^{'}\left(x^{'},t^{'}\right)\int_{0}^{t^{'}}\sigma^{'}\left(x^{'},\tau \right)\varphi^{'}\left(x^{'},\tau \right)\text{d}\tau ,$$ (7)

where **x**′ = **x** / *L* and *t*^'^ = *t* / *T* are the dimensionless space and time coordinates, with *T* = (*λ*_*k*_*λ*_*u*_*φ*_0_*σ*_0_)^−1/2^ (a characteristic time of drug-induced apoptotic cycles; as shown previously [20], this corresponds to the short-term apoptosis time, as the drug uptake reaches significant levels). The dimensionless drug concentration is *σ*^'^ = *σ* / *σ*_0_, and the tumor volume fraction is made dimensionless by *φ*^'^ = *φ* / *φ*_0_. Similarly, the boundary conditions in Eqs 3 and 4 can be nondimensionalized as

$$\sigma^{'}\left(r^{'}=\frac{r_{b}}{L},t^{'}\right)=1\;,\;\text{and}$$ (8)

$$n^{'}\cdot {\nabla^{'}\sigma^{'}|}_{x^{'}\to \infty }\to 0,$$ (9)

with *r*^’^ = *r* / *L*, while the initial conditions for the tumor volume fraction in Eq 5 become

$$\varphi^{'}\left(x^{'},t^{'}=0\right)=1.$$ (10)

Note that the nondimensionalization leaves no parameters in the governing differential equations (Eqs 6 and 7), and only special boundary conditions will introduce biophysical parameters into the model.

Considering the cylindrically symmetric domain surrounding a blood vessel, to which the boundary conditions Eqs 8 and 9 apply, Eq 9 can be further revised to

$${\frac{d\sigma^{'}}{dr^{'}}|}_{r^{'}=\frac{r_{\text{b}}}{L\sqrt{\text{BVF}}}}=0.$$ (11)

Thus, the boundary conditions Eqs 8 and 11 introduce two dimensionless parameters in our numerical analysis: *r*_b_ / *L* and BVF. Numerically, we use a two-step process to integrate the generalized model over time. First, we use *φ*^’^ from the previous time step and solve Eq 6 for the steady-state solution of *σ*^’^ to the first order of Δ*r*, the spatial mesh size. Next, we substitute this *σ*^’^ into Eq 7 and compute *φ*^’^ for the current time step using a backward Euler method [43], which is accurate to the first order of Δ*t*, the numerical time step size.

### Formulation of tumor kill from treatment

By integrating the viable tumor volume fraction at each time point over the cylindrical tissue domain surrounding a blood vessel and affected by the drug diffusion, we calculate *f*_kill_ as the ratio of the killed tumor volume to the total initial tumor volume:

$$f_{\text{kill}}(t)=1-\frac{\frac{2}{\varphi_{0}L^{2}}\int_{r_{b}/L}^{r_{b}/\left(L\sqrt{\text{BVF}}\right)}\varphi \left(r,t\right)rdr}{\left(\frac{1}{\text{BVF}}-1\right)\frac{r_{b}^{2}}{L^{2}}}.$$ (12)

as a function of parameters: *r*_b_, blood volume fraction (BVF), and $L=\sqrt{D/\left(\varphi_{0}\lambda_{\text{u}}\right)}$ (the effective diffusion penetration length of the drug). As shown in **Fig 1**, the model domain is comprised of the space between two concentric cylinders. The inner cylinder has a radius *r*_b_ / *L* in dimensionless units, representing the blood vessel at the center of the domain. With the hypothesis that the substrate supply for any spot in a tissue is supported by the closest blood vessel, we estimate *r*_b_ / (*L* BVF^1/2^) to be the dimensionless radius of the influenced tissue volume of the vessel, where BVF < 1. The influenced tissue volume refers to a specific region of tissue that relies on this blood vessel for supply of oxygen and other essential chemicals.

### Special formulation of *f*_kill_ for time-course tumor measurements

Histology data are not always available for determining values (for parameters BFV, *r*_b_, and *L*) needed for using Eq 12. Thus, we develop an alternative form of *f*_kill_ as a function of another set of experimental parameters, the values of which can be obtained from *in vivo* cytotocixity experiments. By substituting Eq 1 into Eq 2, we have

$$\frac{\partial \varphi }{\partial t}=-\lambda_{\text{k}}\;D\int_{0}^{t}\nabla^{2}\sigma d\tau .$$ (13)

Integrating *φ* in Eq 13 over the total tissue volume *V*, we obtain the changing rate of the tumor volume:

$$\frac{d}{dt}\int_{V}\varphi \;\delta V=-\lambda_{\text{k}}D\int_{0}^{t}(\int_{V}\nabla^{2}\sigma \;\delta V)d\tau .$$ (14)

We denote the tumor volume as *V*_T_; by the convergence theorem, Eq 14 can be rewritten as

$$\frac{dV_{\text{T}}}{dt}=-\lambda_{\text{k}}D\;\int_{0}^{t}(\int_{\partial V}-\frac{\partial \sigma }{\partial n}\delta a)d\tau ,$$ (15)

where ∂*V* represents the boundaries of the total tissue volume in question here, and ∂*σ*/∂*n* is the flux of the drugs across the boundaries. It is safe to hypothesize that the flux of the drugs becomes negligible at the tissue boundaries far away from the blood vessel, and hence the only contribution in the boundary integral we consider is the flux at the boundaries next to the blood vessel. For simplicity, we define

$$F\equiv D\int_{\partial V}-\frac{\partial \sigma }{\partial n}\delta a≅D\int_{r_{\text{b}}}-\frac{\partial \sigma }{\partial n}\delta a.$$ (16)

We have previously demonstrated that *in vitro* porous silicon particles achieve a constant release rate of doxorubicin for up to two weeks at neutral pH [44]. Note that *F* is dependent on nanoparticle size; for the same type of nanoparticle, the bigger the size, the slower is the drug release rate [45]. We thus further hypothesize that the rate of change of flux for the first several days is approximately zero, *dF* / *dt* = 0 (i.e., this initial time period is too short for *F* to change significantly); hence *F* is constant, and we have:

$$\frac{dV_{\text{T}}}{dt}=-\lambda_{\text{k}}\int_{0}^{t}F\;d\tau =-\lambda_{\text{k}}Ft,$$ (17)

which leads to

$$V_{\text{T}}=V_{\text{T},0}-\frac{1}{2}\lambda_{k}Ft^{2},$$ (18)

or equivalently,

$$\frac{V_{\text{T}}}{V_{\text{T},0}}=1-\frac{\lambda_{k}Ft^{2}}{2V_{\text{T},0}}.$$ (19)

As defined previously [19], 1−(*V*_T_/*V*_T,0_) is exactly our definition of *f*_kill_, i.e., the fraction of tumor volume killed from chemotherapy. Hence, we obtain a new mathematical formula for calculating the amount of *f*_kill_ through the delivery method of loaded nano-carriers:

$$f_{\text{kill}}=\frac{F\lambda_{\text{k}}}{2V_{\text{T},0}}t^{2}.$$ (20)

Note that there is a quadratic increase in *f*_kill_ with time, which is consistent as previously observed *in vitro* [20] (cfr. Eq. 4a in that reference).

## Results

### Determination of reference values for model parameters

**Fig 2:**
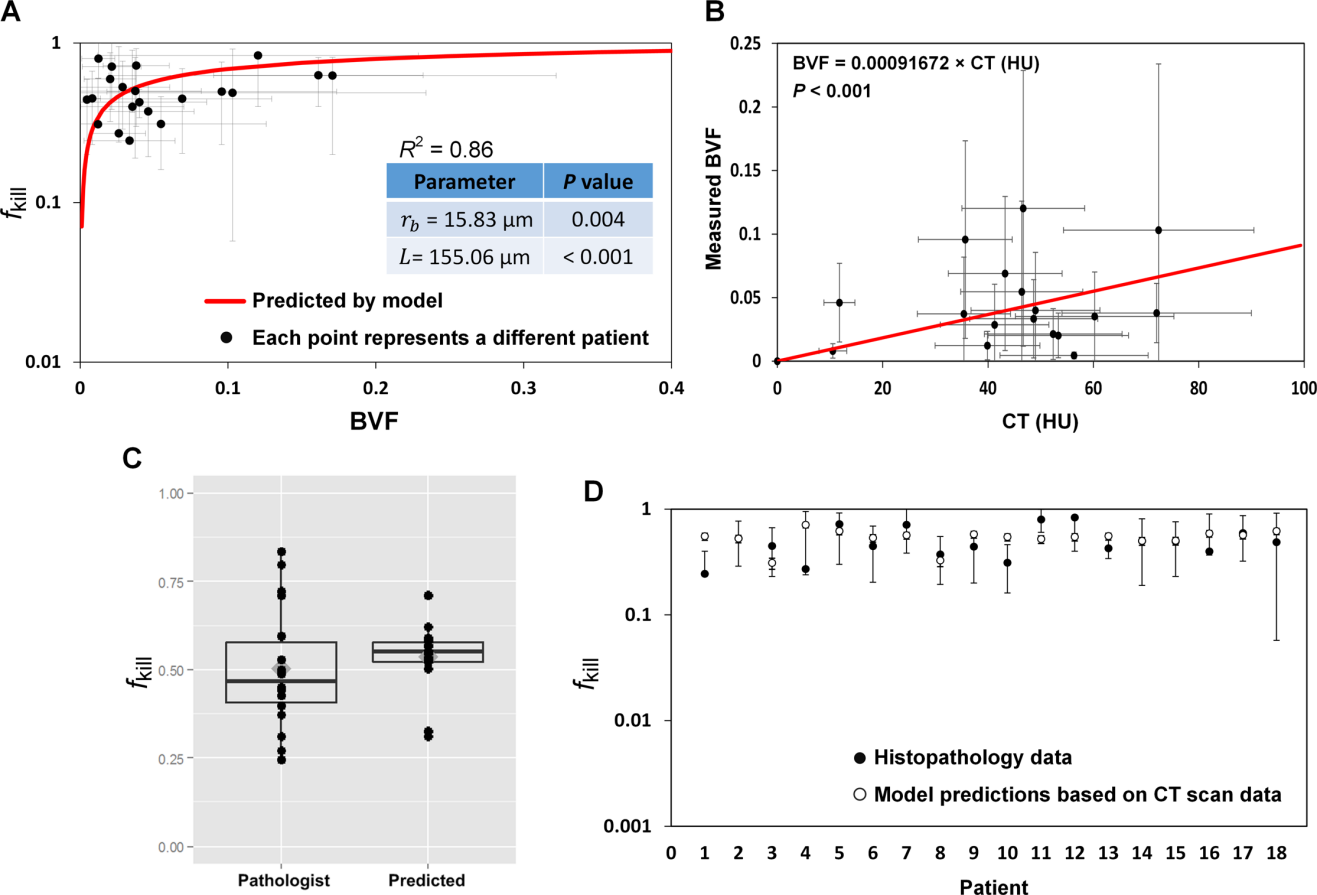
Parameter calibration from patient data demonstrates model predictivity. (*A*) Nonlinear regression analysis of Eq. S2 (coefficient of determination *R*^2^ = 0.86) to the measurements of kill fraction and blood volume fraction BVF from histopathology images of 21 patients with CRC metastatic to liver (standard deviations reflect variability of measured values across 20 slides per patient). Inset: parameter values obtained from fit. (*B*) Linear regression analysis of Hounsfield Unit measurements from pre-treatment arterial-phase contrast-enhanced CT data from 18 patients and blood volume fraction (BVF) measurements from histopathology leads to calibration of BVF parameter (inset). (*C*) Side-by-side boxplots of *f*_kill_ values measured from histopathology and predicted by mathematical model Eq. S2 based on calibration in *A* and *B* (18 data points in each set, symbols). In each boxplot, the thick horizontal line is the median; the box is defined by the 25th and 75th percentiles (lower and upper quartile); the diamond is the mean. A paired t-test at the 0.05 significance level resulted in *P* = 0.44, indicating that the observed difference between the two data sets is not significant. (*D*) Predictions of Eq. S2 (open circles, average relative error ≈ 24%) compared, for each patient, to the direct measurements from histopathology post-treatment and resection (filled circles, with standard deviation of multiple measurements per patient).

For the generalized space- and time-dependent model (Eqs 1 and 2), patient data from multiple time points are usually necessary to determine the model parameters. However, for each patient in our data set obtained from a cohort of 21 patients with CRC metastatic to liver, we had histology data from one time point (after chemotherapy) and contrast CT imaging data from two time points (before and after chemotherapy). Thus, we directly applied Eq. S2 to obtain patient-derived parameter values for BVF, *r*_b_, and *L* (**S1 Text**). Briefly, least-squares fitting of Eq. S2 was performed using Mathematica routine “NonlinearModelFit” [46] to the *f*_kill_ and BVF measured from the histopathology (**S1 Fig** and **S1 Text**). This resulted in estimates of *r*_b_ and *L* parameters which produced the best fit (**Fig 2A**); the values are consistent with published data [47, 48]. Statistically significant *P* values were obtained (inset). Moreover, using regression analysis, we also determined a linear correlation between contrast CT enhancement (in Hounsfield Units) and measured BVF values (**Fig 2B** and **S1 Text**). Standard deviations for CT data reflect 25% variability in physiology and contrast-injection protocols across patients, and was estimated by calculating standard deviation of CT measurements taken within the patient aorta. CT measurements reflect perfusion of tissue, which relies on the volume fraction of blood vessels, hence resulting in *P* < 0.001. The linear correlation (**Fig 2B**) was used to inform model Eq. S2 directly from pre-treatment CT data to predict *f*_kill_ for each patient. The results in **Fig 2C** show that there is no statistically significant difference between the predictions obtained from the linear correlation and direct measurements of kill from histopathology of the resected tumors. Lastly, the predicted *f*_kill_ based on CT scans *prior to treatment* (**Fig 2D**, open circles) matched the measured *f*_kill_ (filled circles) *after treatment* with an average relative error of ≈ 24%. Hence, we used the obtained, verified parameter values (for BVF, *r*_b_, and *L*) as reference values for subsequent simulation studies.

### Parameter analysis of the generalized model

**Fig 3:**
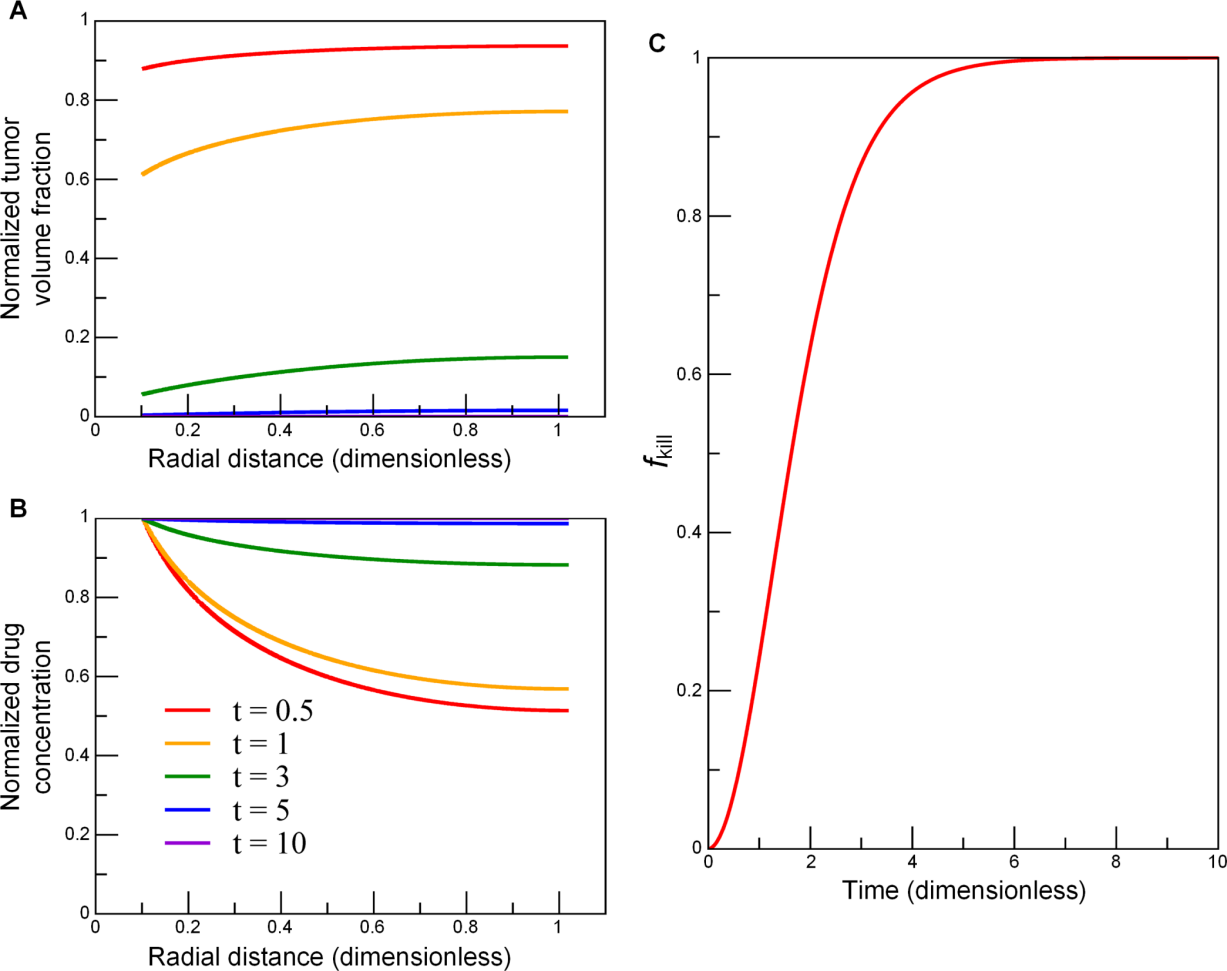
Numerical simulations of the general integro-differential model (Eqs 6 and 7) in a cylindrically symmetric domain. As cell kill ensues over several cell cycles, (*A*) successive cell layers next to the blood vessel (*r* = *r*_b_) die out, i.e., tumor volume fraction *φ* decreases; (*B*) local drug concentration *σ* increases due to an enhancement of drug penetration; and (*C*) cell kill accelerates further from the vessel and deep into the tumor. Input parameters: *r*_b_ / *L* = 0.102 and BVF = 0.01. The duration of the entire simulation was 10 (*λ*_*k*_*λ*_*u*_*φ*_0_*σ*_0_)^−1/2^, where time unit is a characteristic cell apoptosis time. Drug concentration and tumor volume fraction were normalized by their initial values, and radial distance by the diffusion penetration distance *L*. The fraction of tumor kill *f*_kill_ is calculated from Eq 12 (**Methods**).

We performed a set of simulations with the generalized time- and space-dependent model in a cylindrically symmetric domain surrounding a blood vessel. Model parameter values were set as follows (**Fig 2A**): *L* = 155.06 μm and *r*_b_ = 15.83 μm, giving *r*_b_/*L* = 0.102; we also set BVF = 10^−2^ as the reference value for the simulations, as measured BVF values ranged approximately from 10^−3^ to 10^−1^. We ran the model (Eqs 6 and 7) for 10 drug-induced apoptotic cycles. To examine the impact of each parameter on *f*_kill_, we further simulated nine parameter-variation combinations, using three *r*_b_/*L* values, i.e., 0.05, 0.1, 0.5, paired with three BVF values, i.e., 0.005, 0.01, 0.05. **Fig 3** shows the numerical results of the model. In the presence of a boundary condition *σ*_0_ at the vessel wall (*r* = *r*_b_), successive cell layers next to the blood vessel die out (**Fig 3A**) due to enhancement of drug penetration (**Fig 3B**), in turn leading to accelerated cell kill (**Fig 3C**). As cell kill occurs, tumor volume fraction *φ* decreases, leading to an increase in local drug concentration *σ* (because dead cells no longer take drugs), and thus accelerating cell kill in the locations further away from the vessel and deep into the tumor.

**Fig 4:**
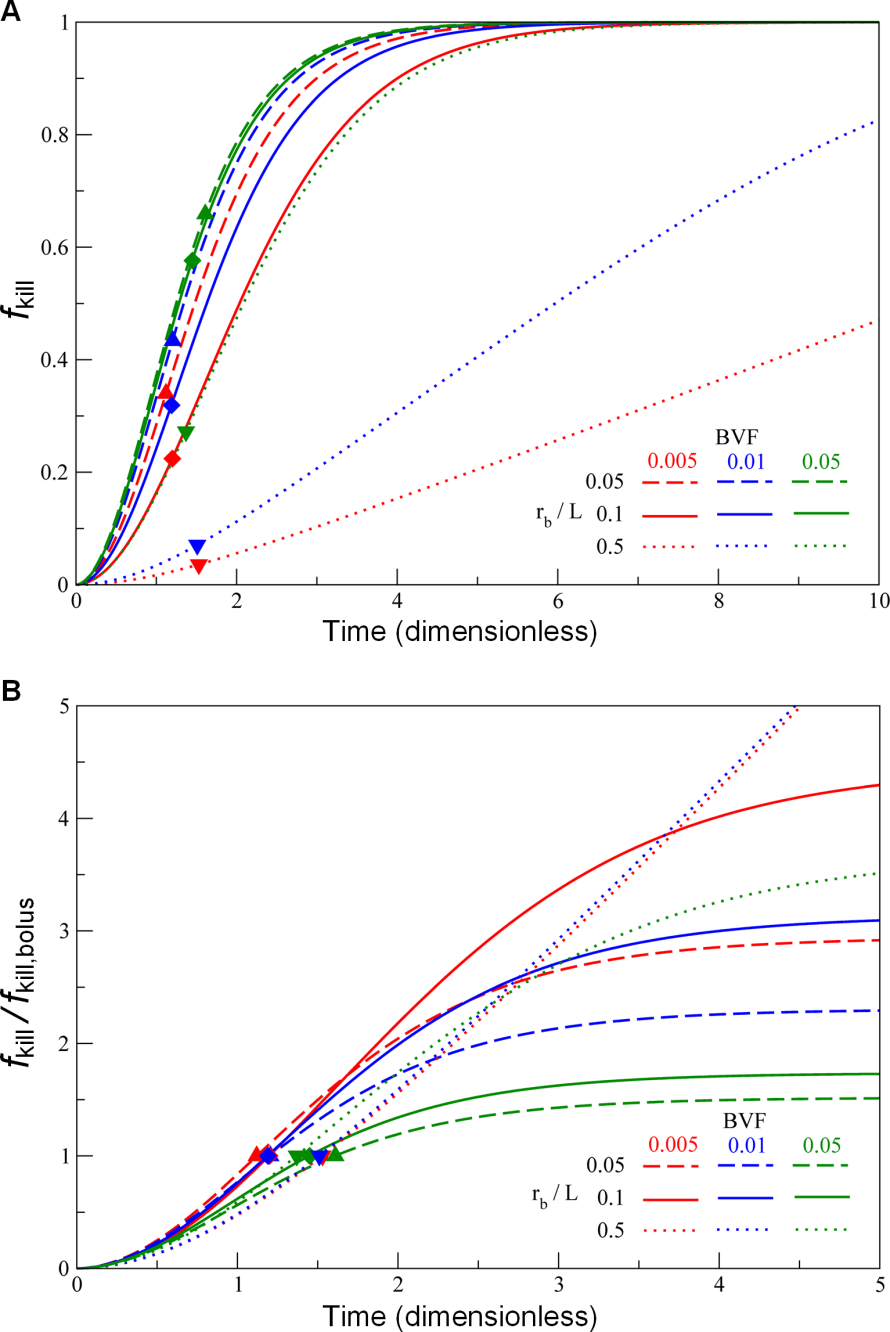
Drug-loaded nano-carriers lead to cell-kill enhancement over bolus delivery. (*A*) Time-evolution curves of chemotherapeutic efficacy *f*_kill_ (Eq 12) of nano-carriers releasing drug compared to the estimated efficacy (symbols) of conventional chemotherapy (Eq. S2), for parameter values: *r*_b_ / *L* = 0.05 (dashed curves, upper triangles), 0.1 (solid curves, diamonds), and 0.5 (dotted curves, lower triangles), paired with BVF = 0.005 (red curves and symbols), 0.01 (blue curves and symbols), and 0.05 (green curves and symbols). (*B*) Same as (*A*), but normalized to the corresponding bolus values of tumor kill, *f*_kill,bolus_.

**Fig 4A** shows the temporal evolution curves of *f*_kill_ calculated from Eq 12 by varying the parameters *r*_b_/*L* and BVF, representing conditions where drug-loaded nano-carriers are employed, as well as the estimates using our simplified “bolus” model (i.e., Eq. S2). The results indicate that the “bolus” killing ratios are readily achieved by drug-loaded nano-carriers after 1 or 2 cell cycles. To estimate the benefits of drug release by the nano-carriers over a longer period of time, we normalized the *f*_kill_ curves using their corresponding bolus *f*_kill_ values (**Fig 4B**). The results suggest that we may achieve 2- to 4-fold of the bolus killing ratios if the drug release from nano-carriers administration lasts for 3 or 4 apoptotic cycles. However, for large BVF values (representing highly vascularized tumors), cell killing effects from both methods of delivery are roughly equivalent. This is expected because the majority of the tumor cells are killed within just one or two apoptotic cell cycles; a 50% increase in tumor kill is nevertheless expected from loaded nano-carriers releasing over a longer period of time. This suggests an alternative strategy to improve chemotherapeutic efficacy by promoting or normalizing angiogenesis at the target site before administrating chemotherapy drugs [2, 49–51], or by promoting perfusion by other means such as mild hyperthermia [52], both of which would lead to an increase in BVF. We also note that, in our simulations, the domain size (i.e., the radius of the outer cylinder) was determined by *r*_b_ / (*L* BVF^1/2^). Thus, a larger *r*_b_ / *L* or a smaller BVF represented a larger tissue volume relying on the blood vessel for drug transport, and hence required a longer time to achieve the same *f*_kill_.

### Experimental validation

#### Ethics statement

The animal studies were performed in accordance with the guidelines of the Animal Welfare Act and the Guide for the Care and Use of Laboratory Animals following protocols approved by the Institutional Animal Care and Use Committee (IACUC).

#### Cytotoxicity experiments: Comparison of free and nano-particle-based drug delivery *in vivo*

A total of 40 six-week-old female BALB/c mice were obtained from Charles River Laboratories and kept in a specific pathogen-free facility at the Houston Methodist Research Institute. On day zero, mice were inoculated with 5 x 10^4^ green fluorescent protein (GFP)-labeled murine 4T1 cells in their inguinal mammary fat pad. Breast tumors were allowed to grow for two weeks to reach a volume *V*_T,0_ = 100–200 mm^3^. Mice were then randomly placed into four groups (10 mice per group) and administered intravenously beginning on day 14 according to a predefined protocol. The four groups were: **i)** control: phosphate buffered saline (PBS), administered twice a week; **ii)** free doxorubicin (3 mg/kg, i.v.), administered twice a week; **iii)** 1.0 μm porous silicon particle loaded with chemotherapy drug (iNPG/pDox 1.0, 6 mg/kg, i.v.) [53], administered once a week; and **iv)** 2.6 μm porous silicon particle loaded with chemotherapy drug (iNPG/pDox 2.6, 6 mg/kg, i.v.), administered once a week. Tumor volume was measured for each mouse on days 14, 17, 21, 25, 28, and 31 after tumor cell injection. Mice were sacrificed on day 31 via CO_2_ asphyxiation and tumors were removed. For comparison with model predictions (i.e., Eq 20), the tumor volume measurements were normalized across the four treatment groups to the measurements from the PBS control group and to the initial tumor volume, and for each tumor, *f*_kill_ was calculated as 1 minus the normalized tumor volume.

#### Model predictions

**Fig 5:**
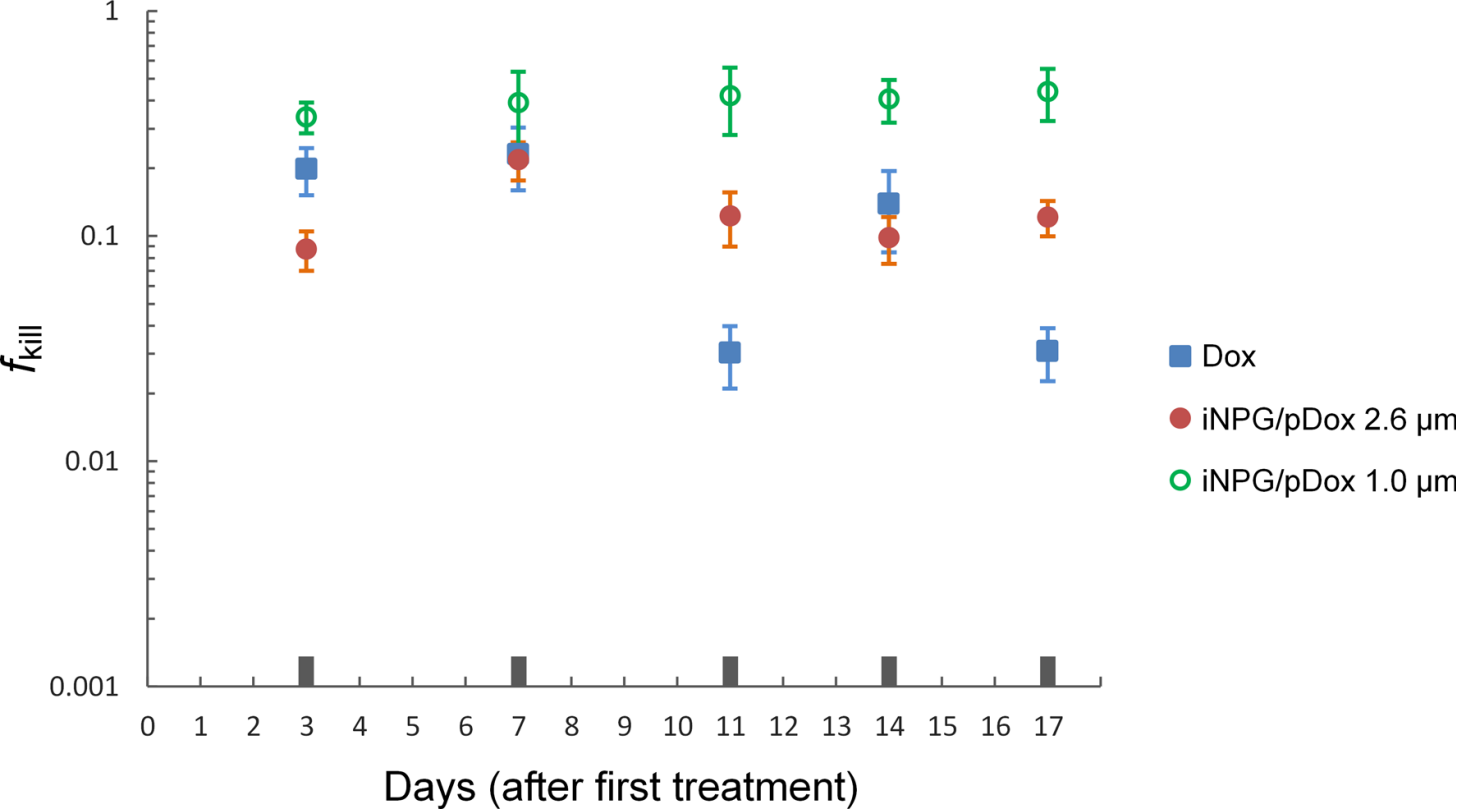
Testing the efficacy of drug-loaded nano-carriers in mice. Comparison of fraction of tumor killed measured across three different treatment BALB/c mice groups (n = 10 per group) over a period of 17 days (from day 14 to day 31 after 4T1 tumor cell inoculation, see **Methods**) showing a roughly 3-fold increase in kill from nano-vectored drug vs. free drug. At each time point, tumor volume measurements from the three drug treatment groups were first normalized to the measurement from the control (PBS) group (no drug treatment), and then to the initial tumor volume for each group; *f*_kill_ was then calculated as (1 –normalized tumor volume).

From the time-evolution of *f*_kill_ for the three groups of BALB/c mice (**Fig 5**), it is evident that, after roughly three days of first treatment with rapid growth, *f*_kill_ remains approximately constant until the end of the experiments (*f*_kill_ = 0 at the onset of the treatment on day zero). This rapid growth of the fraction of dead cells is consistent with the quadratic time-dependence predicted by model Eq 20. The measured tumor kill from nano-vectors is about 0.5, and roughly 3 times that from free drug, in excellent agreement with the model predictions of a 2–4 fold increase in kill depending on the parameter values (cfr. **Fig 4B**). From the experimental protocol, we know that the total amount of drug released by the particles is *F*∙*t* ≈ 1.2∙10^−4^ g for a typical mouse weight of 20 g [44, 54–56]. We then analyzed the tumor growth curves (**S3 Fig**) and estimated the approximate (linear) growth rates. For example, the controls were found to grow at an average rate of Λ ≈ 70 mm^3^ / day (proliferation only; no death), while the tumors in mice treated with iNPG/pDox 1.0 μm grow at a rate of ≈ 35 mm^3^ / day (net outcome of proliferation minus death rates). The latter result produces a net death rate for the iNPG/pDox 1.0 μm treated tumors of Λ_k_ ≈ 35 mm^3^ / day, which, since the specific rate of kill (per molecule of drug) is *λ*_k_ = Λ_k_ / (*Ft*), gives, together with Eq 20, an estimate for $t_{\text{kill}}=\frac{2V_{0}}{\Lambda_{\text{k}}}\cdot f_{\text{kill}}\approx 4\;\text{days}$ (corresponding to *f*_kill_ ≈ 0.5 and *V*_0_ = 130 mm^3^), *in excellent agreement with the observed time to plateau of the cell kill reported in the experiments* (**Fig 5**). Note that subsequent treatments in the protocol are relatively irrelevant, as the amount of drug used is the same whereas the tumors have already grown by one order of magnitude by the time the second treatment is applied (i.e., one week).

## Discussion

A fundamental principle of oncologic therapy is that cancer cell kill must be absolute to achieve a cure. For patients with metastatic cancer, conventional systemic chemotherapy alone does not eradicate all of the disease [57]. This therapeutic challenge is evident in clinical practice with both cytotoxic and cytostatic therapies, and our mathematical model of cellular response to chemotherapy highlights a physical mechanism for this clinical observation [19, 22]. Indeed, in our original report of this mathematical description of cancer cell kill due to local drug concentrations, we found that complete eradication of the cells was impossible using current methods to infuse drugs intravenously [23]. Here, we have confirmed this finding in a larger dataset of patients with colorectal liver metastases. Notably, we find that the pathological response to chemotherapy is heterogeneous within a given tumor and that the local physical properties of the tumor describe this response. This is significant in understanding therapeutic resistance, suggesting that the physical microenvironment naturally selects cancer cells that reside in areas with poor drug penetration.

This observation of heterogeneous response inspired a concept of improving drug delivery using nanoparticles that accumulate within the tumor, delivering sustained local release of drug to the cancer cells. We simulated this concept by generalizing our mechanistic model and applying it to the same patient group with colorectal liver metastases. This generalized model now accounts for the spatial *and* temporal heterogeneities of drug dosing, which builds on our prior work [19, 20]. From the parameter analysis (**Figs 3** and **4**), we find that an extended period of treatment achieves a better treatment outcome (i.e., killing more cancer cells). More importantly, we found that the delivery of systemic chemotherapy using these nanoparticles would have enhanced cell kill by a factor of 2 to 4 over the standard therapy that the patients actually received (**Fig 4B**). However, this strategy may not be feasible in practice due to the constraint of tolerable cytotoxicity to healthy cells; further investigation into tumor-targeted nano-carriers may be necessary to realize the results in patients that our model predicts.

In our model, for the case of cells that stay further away from the blood vessel, the reduction of cell death is only due to lowered local drug concentration. Indeed, these cells may also experience a change in other microenvironmental conditions (such as hypoxia and nutrient deprivation), which can lead to reduced proliferation rates and therefore reduced sensitivity to cytotoxic drugs. To address this issue, we could add oxygen concentration as a parameter to the model, as demonstrated in our prior work [37]. However, in light of the insight we gained from our model for enhanced drug penetration (**Fig 3**), oxygen may also penetrate deeper into the surrounding tissue because dead cells no longer uptake oxygen. Therefore, our assumption of a fixed cell sensitivity to drugs may remain valid even without taking oxygen into consideration. In our ongoing work, we are validating our model results shown in **Fig 3** with in vivo experiments.

Another observation from our model is that a larger BVF results in a higher tumor killing ratio. One may envision extending our generalized model to other therapeutic strategies that aim to improve efficacy through enhanced drug delivery by increasing BVF. These strategies include, but are not limited to, vascular normalization [2], metronomic dosing [58], and stromal targeting [29, 30]. Each of these physical sciences-based therapeutic strategies could be optimized through our predictive mathematical model.

As an experimental demonstration of this concept of drug-loaded nano-carriers, we used a porous silicon particle delivery system loaded with chemotherapy in a mouse model of breast cancer. We have found that these particles localize within tumors [59], likely due to the geometry of the particles. As predicted by our model, we measured a 3.5-fold difference in tumor growth rate in the mice tumors treated with drug-loaded nanoparticles compared to the tumors treated with systemic delivery of doxorubicin (**Fig 5**). This experimental confirmation of the model results provides rationale to translate these findings and concepts to patients.

In the future, additional layers of complexity involving other factors or physiological barriers, such as tumor cell regrowth or proliferation that repopulates the killed region, effect of cell to cell contact, and effect of chemotherapy on the tumor vasculature [25, 37, 38], can be added to the model in an effort to improve the accuracy of model predictions. Importantly, this model applies to any systemic agent, including immunotherapy. For example, the model may be generalized even more to account for cancer cell kill by the immune system, accounting for the physical barriers to immune cell infiltration into the cancer. Moreover, through the use of non-invasive characterization of transport prior to therapy using diagnostic CT or MRI imaging, as well as the biological characterization of molecular targets for an individual tumor [60], one could optimize both drug delivery and therapeutic selection for a given patient. This biophysical characterization and prediction strategy would complement genetically-based, patient-specific cancer therapy methods by individualizing drug administration regimens.

## Supporting Information

S1 Text Supplementary material. (DOCX)

S1 Fig Example of measurements from histopathological specimens. (*A*) A portal triad in normal liver. (*B*) Example of a histologic section from one patient. (*C*) Segmentation of the histologic section *B* for calculation of the fraction of dead tumor area: dead tumor (red); live tumor (blue); no tumor (green).
(TIF)

S2 Fig Comparisons of cumulative distributions of measured *f*_kill_ in tissue. Data were obtained from histopathology images of 21 patients with CRC metastatic to liver. Measurements by image segmentation using the GIMP software (blue); standard clinical assessment by the pathologist at MDACC (red). (*A*) Measurements of *f*_kill_ by software vs. by pathologist. (*B*) Measurements of *f*_kill_ by software and shifted to the right by 0.108 vs. by pathologist.
(TIF)

S3 Fig Measurements of tumor volume. Four treatment groups: PBS (control), free doxorubicin, 1.0 μm porous silicon particle loaded with chemotherapy drug (iNPG/pDox 1.0), and 2.6 μm porous silicon particle loaded with chemotherapy drug (iNPG/pDox 2.6). Data were measured on days 0, 3, 7, 11, 14, and 17 after first treatment.
(TIF)

## References

[1] Junttila MR, de Sauvage FJ. Influence of tumour micro-environment heterogeneity on therapeutic response. Nature. 2013;501(7467):346–54. Epub 2013/09/21. 10.1038/nature12626 24048067

[2] Goel S, Wong AH, Jain RK. Vascular normalization as a therapeutic strategy for malignant and nonmalignant disease. Cold Spring Harbor perspectives in medicine. 2012;2(3):a006486 Epub 2012/03/07. 10.1101/cshperspect.a006486 22393532 PMC3282493

[3] Ferrari M. Frontiers in cancer nanomedicine: directing mass transport through biological barriers. Trends in biotechnology. 2010;28(4):181–8. Epub 2010/01/19. 10.1016/j.tibtech.2009.12.007 20079548 PMC2843761

[4] Ozdemir BC, Pentcheva-Hoang T, Carstens JL, Zheng X, Wu CC, Simpson TR, et al Depletion of carcinoma-associated fibroblasts and fibrosis induces immunosuppression and accelerates pancreas cancer with reduced survival. Cancer Cell. 2014;25(6):719–34. 10.1016/j.ccr.2014.04.005 24856586 PMC4180632

[5] Rhim AD, Oberstein PE, Thomas DH, Mirek ET, Palermo CF, Sastra SA, et al Stromal elements act to restrain, rather than support, pancreatic ductal adenocarcinoma. Cancer Cell. 2014;25(6):735–47. 10.1016/j.ccr.2014.04.021 24856585 PMC4096698

[6] Kim M, Gillies RJ, Rejniak KA. Current advances in mathematical modeling of anti-cancer drug penetration into tumor tissues. Frontiers in Oncology. 2013;3. 10.3389/fonc.2013.00278 PMC3831268 24303366

[7] Li M, Al-Jamal KT, Kostarelos K, Reineke J. Physiologically based pharmacokinetic modeling of nanoparticles. ACS nano. 2010;4(11):6303–17. Epub 2010/10/16. 10.1021/nn1018818 20945925

[8] Michor F, Beal K. Improving cancer treatment via mathematical modeling: Surmounting the challenges is worth the effort. Cell. 2015;163(5):1059–63. 10.1016/j.cell.2015.11.002 26590416 PMC4676401

[9] Deisboeck TS, Wang Z, Macklin P, Cristini V. Multiscale cancer modeling. Annual review of biomedical engineering. 2011;13:127–55. Epub 2011/05/03. 10.1146/annurev-bioeng-071910-124729 21529163 PMC3883359

[10] Wang Z, Butner JD, Cristini V, Deisboeck TS. Integrated PK-PD and agent-based modeling in oncology. Journal of pharmacokinetics and pharmacodynamics. 2015;42(2):179–89. Epub 2015/01/16. 10.1007/s10928-015-9403-7 25588379 PMC4985529

[11] Basanta D, Strand DW, Lukner RB, Franco OE, Cliffel DE, Ayala GE, et al The role of transforming growth factor-beta-mediated tumor-stroma interactions in prostate cancer progression: an integrative approach. Cancer Res. 2009;69(17):7111–20. Epub 2009/08/27. 10.1158/0008-5472.CAN-08-3957 19706777 PMC2748342

[12] Venkatasubramanian R, Arenas RB, Henson MA, Forbes NS. Mechanistic modelling of dynamic MRI data predicts that tumour heterogeneity decreases therapeutic response. British journal of cancer. 2010;103(4):486–97. Epub 2010/07/16. 10.1038/sj.bjc.6605773 20628390 PMC2939778

[13] Zhao B, Hemann MT, Lauffenburger DA. Intratumor heterogeneity alters most effective drugs in designed combinations. Proc Natl Acad Sci U S A. 2014;111(29):10773–8. Epub 2014/07/09. 10.1073/pnas.1323934111 25002493 PMC4115561

[14] Haeno H, Gonen M, Davis MB, Herman JM, Iacobuzio-Donahue CA, Michor F. Computational modeling of pancreatic cancer reveals kinetics of metastasis suggesting optimum treatment strategies. Cell. 2012;148(1–2):362–75. Epub 2012/01/24. 10.1016/j.cell.2011.11.060 22265421 PMC3289413

[15] Weis JA, Miga MI, Arlinghaus LR, Li X, Abramson V, Chakravarthy AB, et al Predicting the Response of Breast Cancer to Neoadjuvant Therapy Using a Mechanically Coupled Reaction-Diffusion Model. Cancer Res. 2015;75(22):4697–707. Epub 2015/09/04. 10.1158/0008-5472.CAN-14-2945 26333809 PMC4651826

[16] Weis JA, Miga MI, Arlinghaus LR, Li X, Chakravarthy AB, Abramson V, et al A mechanically coupled reaction-diffusion model for predicting the response of breast tumors to neoadjuvant chemotherapy. Physics in medicine and biology. 2013;58(17):5851–66. Epub 2013/08/08. 10.1088/0031-9155/58/17/5851 23920113 PMC3791925

[17] Gatenby RA, Silva AS, Gillies RJ, Frieden BR. Adaptive therapy. Cancer Res. 2009;69(11):4894–903. Epub 2009/06/03. 10.1158/0008-5472.CAN-08-3658 19487300 PMC3728826

[18] Thurber GM, Yang KS, Reiner T, Kohler RH, Sorger P, Mitchison T, et al Single-cell and subcellular pharmacokinetic imaging allows insight into drug action in vivo. Nature communications. 2013;4:1504 Epub 2013/02/21. 10.1038/ncomms2506 23422672 PMC3579506

[19] Pascal J, Bearer EL, Wang Z, Koay EJ, Curley SA, Cristini V. Mechanistic patient-specific predictive correlation of tumor drug response with microenvironment and perfusion measurements. Proc Natl Acad Sci U S A. 2013;110(35):14266–71. 10.1073/pnas.1300619110 23940372 PMC3761643

[20] Pascal J, Ashley CE, Wang Z, Brocato TA, Butner JD, Carnes EC, et al Mechanistic modeling identifies drug-uptake history as predictor of tumor drug resistance and nano-carrier-mediated response. ACS nano. 2013;7(12):11174–82. Epub 2013/11/06. 10.1021/nn4048974 24187963 PMC3891887

[21] Das H, Wang Z, Niazi MK, Aggarwal R, Lu J, Kanji S, et al Impact of diffusion barriers to small cytotoxic molecules on the efficacy of immunotherapy in breast cancer. PloS one. 2013;8(4):e61398 Epub 2013/04/27. 10.1371/journal.pone.0061398 23620747 PMC3631240

[22] Koay EJ, Baio FE, Ondari A, Truty MJ, Cristini V, Thomas RM, et al Intra-tumoral heterogeneity of gemcitabine delivery and mass transport in human pancreatic cancer. Physical biology. 2014;11(6):065002 Epub 2014/11/27. 10.1088/1478-3975/11/6/065002 25427073 PMC4266401

[23] Koay EJ, Truty MJ, Cristini V, Thomas RM, Chen R, Chatterjee D, et al Transport properties of pancreatic cancer describe gemcitabine delivery and response. The Journal of clinical investigation. 2014;124(4):1525–36. Epub 2014/03/13. 10.1172/JCI73455 24614108 PMC3973100

[24] Saggar JK, Fung AS, Patel KJ, Tannock IF. Use of molecular biomarkers to quantify the spatial distribution of effects of anticancer drugs in solid tumors. Mol Cancer Ther. 2013;12(4):542–52. 10.1158/1535-7163.MCT-12-0967 23348047

[25] Tannock IF. Tumor physiology and drug resistance. Cancer metastasis reviews. 2001;20(1–2):123–32. Epub 2002/02/08. 11831640 10.1023/a:1013125027697

[26] Chauhan VP, Martin JD, Liu H, Lacorre DA, Jain SR, Kozin SV, et al Angiotensin inhibition enhances drug delivery and potentiates chemotherapy by decompressing tumour blood vessels. Nature communications. 2013;4:2516 10.1038/ncomms3516 24084631 PMC3806395

[27] Minchinton AI, Tannock IF. Drug penetration in solid tumours. Nat Rev Cancer. 2006;6(8):583–92. 16862189 10.1038/nrc1893

[28] Tredan O, Galmarini CM, Patel K, Tannock IF. Drug resistance and the solid tumor microenvironment. Journal of the National Cancer Institute. 2007;99(19):1441–54. Epub 2007/09/27. 17895480 10.1093/jnci/djm135

[29] Olive KP, Jacobetz MA, Davidson CJ, Gopinathan A, McIntyre D, Honess D, et al Inhibition of Hedgehog signaling enhances delivery of chemotherapy in a mouse model of pancreatic cancer. Science. 2009;324(5933):1457–61. Epub 2009/05/23. 10.1126/science.1171362 19460966 PMC2998180

[30] Provenzano PP, Cuevas C, Chang AE, Goel VK, Von Hoff DD, Hingorani SR. Enzymatic targeting of the stroma ablates physical barriers to treatment of pancreatic ductal adenocarcinoma. Cancer cell. 2012;21(3):418–29. Epub 2012/03/24. 10.1016/j.ccr.2012.01.007 22439937 PMC3371414

[31] Edgerton ME, Chuang YL, Macklin P, Yang W, Bearer EL, Cristini V. A novel, patient-specific mathematical pathology approach for assessment of surgical volume: application to ductal carcinoma in situ of the breast. Anal Cell Pathol. 2011;34(5):247–63. 10.3233/ACP-2011-0019 PMC3613121 21988888

[32] Shen H, Rodriguez-Aguayo C, Xu R, Gonzalez-Villasana V, Mai J, Huang Y, et al Enhancing chemotherapy response with sustained EphA2 silencing using multistage vector delivery. Clin Cancer Res. 2013;19(7):1806–15. Epub 2013/02/07. 10.1158/1078-0432.CCR-12-2764 23386691 PMC3618564

[33] Tanaka T, Mangala LS, Vivas-Mejia PE, Nieves-Alicea R, Mann AP, Mora E, et al Sustained small interfering RNA delivery by mesoporous silicon particles. Cancer Res. 2010;70(9):3687–96. Epub 2010/05/01. 10.1158/0008-5472.CAN-09-3931 20430760 PMC3202607

[34] Xu R, Huang Y, Mai J, Zhang G, Guo X, Xia X, et al Multistage vectored siRNA targeting ataxia-telangiectasia mutated for breast cancer therapy. Small. 2013;9(9–10):1799–808. Epub 2013/01/08. 10.1002/smll.201201510 23293085 PMC3842236

[35] Kim B, Han G, Toley BJ, Kim CK, Rotello VM, Forbes NS. Tuning payload delivery in tumour cylindroids using gold nanoparticles. Nature nanotechnology. 2010;5(6):465–72. Epub 2010/04/13. 10.1038/nnano.2010.58 20383126 PMC2881185

[36] Lee T-R, Choi M, Kopacz AM, Yun S-H, Liu WK, Decuzzi P. On the near-wall accumulation of injectable particles in the microcirculation: smaller is not better. Scientific Reports. 2013;3:2079 http://www.nature.com/articles/srep02079#supplementary-information. 10.1038/srep02079 23801070 PMC3693098

[37] Frieboes HB, Wu M, Lowengrub J, Decuzzi P, Cristini V. A computational model for predicting nanoparticle accumulation in tumor vasculature. PloS one. 2013;8(2):e56876 Epub 2013/03/08. 10.1371/journal.pone.0056876 23468887 PMC3585411

[38] van de Ven AL, Wu M, Lowengrub J, McDougall SR, Chaplain MA, Cristini V, et al Integrated intravital microscopy and mathematical modeling to optimize nanotherapeutics delivery to tumors. AIP Adv. 2012;2(1):11208 Epub 2012/04/11. 22489278 10.1063/1.3699060 PMC3321519

[39] Sorrell I, Shipley RJ, Hearnden V, Colley HE, Thornhill MH, Murdoch C, et al Combined mathematical modelling and experimentation to predict polymersome uptake by oral cancer cells. Nanomedicine: nanotechnology, biology, and medicine. 2014;10(2):339–48. Epub 2013/09/17. 10.1016/j.nano.2013.08.013 24036098

[40] Stapleton S, Milosevic M, Allen C, Zheng J, Dunne M, Yeung I, et al A mathematical model of the enhanced permeability and retention effect for liposome transport in solid tumors. PloS one. 2013;8(12):e81157 Epub 2013/12/07. 10.1371/journal.pone.0081157 24312530 PMC3846845

[41] Kerr DJ, Kerr AM, Freshney RI, Kaye SB. Comparative intracellular uptake of adriamycin and 4'-deoxydoxorubicin by non-small cell lung tumor cells in culture and its relationship to cell survival. Biochemical pharmacology. 1986;35(16):2817–23. Epub 1986/08/15. 3741470 10.1016/0006-2952(86)90195-4

[42] Gigli M, Rasoanaivo TW, Millot JM, Jeannesson P, Rizzo V, Jardillier JC, et al Correlation between growth inhibition and intranuclear doxorubicin and 4'-deoxy-4'-iododoxorubicin quantitated in living K562 cells by microspectrofluorometry. Cancer Res. 1989;49(3):560–4. Epub 1989/02/01. 2910478

[43] Chapra S, Canale R. Numerical Methods for Engineers. 6th ed. New York, NY: McGraw-Hill Science/Engineering/Math; 2009.

[44] Shen H, Ferrari M, Deng X, Zhang G. Compositions and methods of treating therapy resistant cancer and uses thereof. Google Patents; 2014.

[45] Golomb G, Fisher P. The relationship between drug release rate, particle size and swelling of silicone matrices. Journal of controlled release: official journal of the Controlled Release Society. 1990;12(2):121–32.

[46] Wolfram Research. Mathematica, Version 8.0, Mathematics and Algorithms. http://www.wolfram.com/learningcenter/tutorialcollection/MathematicsAndAlgorithms/MathematicsAndAlgorithms.pdf2008.

[47] Muraca M. Methods in Biliary Research. United Stated of America: CRC Press; 1994.

[48] Wiedeman MP. Dimensions of blood vessels from distributing artery to collecting vein. Circ Res. 1963;12:375–8. 14000509 10.1161/01.res.12.4.375

[49] Huang Y, Goel S, Duda DG, Fukumura D, Jain RK. Vascular normalization as an emerging strategy to enhance cancer immunotherapy. Cancer research. 2013;73(10):2943–8. 10.1158/0008-5472.CAN-12-4354 23440426 PMC3655127

[50] Huang Y, Stylianopoulos T, Duda DG, Fukumura D, Jain RK. Benefits of vascular normalization are dose and time dependent—letter. Cancer research. 2013;73(23):7144–6. 10.1158/0008-5472.CAN-13-1989 24265277 PMC3876035

[51] Jain RK, Tong RT, Munn LL. Effect of vascular normalization by antiangiogenic therapy on interstitial hypertension, peritumor edema, and lymphatic metastasis: insights from a mathematical model. Cancer research. 2007;67(6):2729–35. 17363594 10.1158/0008-5472.CAN-06-4102 PMC3022341

[52] Kirui DK, Koay EJ, Guo X, Cristini V, Shen H, Ferrari M. Tumor vascular permeabilization using localized mild hyperthermia to improve macromolecule transport. Nanomedicine: nanotechnology, biology, and medicine. 2014;10(7):1487–96. Epub 2013/11/23. 10.1016/j.nano.2013.11.001 PMC4025989 24262998

[53] Xu R, Zhang G, Mai J, Deng X, Segura-Ibarra V, Wu S, et al An injectable nanoparticle generator enhances delivery of cancer therapeutics. Nature biotechnology. 2016;34(4)414–8. Epub 2016/03/15. 10.1038/nbt.3506 26974511 PMC5070674

[54] Dai C-L, Xiong H-Y, Tang L-F, Zhang X, Liang Y-J, Zeng M-S, et al Tetrandrine achieved plasma concentrations capable of reversing MDR in vitro and had no apparent effect on doxorubicin pharmacokinetics in mice. Cancer chemotherapy and pharmacology. 2007;60(5):741–50. 17273824 10.1007/s00280-007-0420-0

[55] Lu WL, Qi XR, Zhang Q, Li RY, Wang GL, Zhang RJ, et al A pegylated liposomal platform: pharmacokinetics, pharmacodynamics, and toxicity in mice using doxorubicin as a model drug. J Pharmacol Sci. 2004;95(3):381–9. 15272215 10.1254/jphs.fpj04001x

[56] Richly H, Grubert M, Scheulen ME, Hilger RA. Plasma and cellular pharmacokinetics of doxorubicin after intravenous infusion of Caelyx/Doxil in patients with hematological tumors: Int J Clin Pharmacol Ther. 2009 1;47(1):55–7. 19203539 10.5414/cpp47055

[57] Gennari A, Stockler M, Puntoni M, Sormani M, Nanni O, Amadori D, et al Duration of chemotherapy for metastatic breast cancer: a systematic review and meta-analysis of randomized clinical trials. Journal of clinical oncology: official journal of the American Society of Clinical Oncology. 2011;29(16):2144–9. Epub 2011/04/06. 21464403 10.1200/JCO.2010.31.5374

[58] Pasquier E, Kavallaris M, Andre N. Metronomic chemotherapy: new rationale for new directions. Nature reviews Clinical oncology. 2010;7(8):455–65. Epub 2010/06/10. 10.1038/nrclinonc.2010.82 20531380

[59] Decuzzi P, Godin B, Tanaka T, Lee SY, Chiappini C, Liu X, et al Size and shape effects in the biodistribution of intravascularly injected particles. Journal of controlled release: official journal of the Controlled Release Society. 2010;141(3):320–7. Epub 2009/10/31. 10.1016/j.jconrel.2009.10.014 19874859

[60] Wang Z, Deisboeck TS. Mathematical modeling in cancer drug discovery. Drug discovery today. 2014;19(2):145–50. Epub 2013/07/09. 10.1016/j.drudis.2013.06.015 23831857

