# Supplementary material for: Theory and Experimental Validation of a Spatio-temporal Model of Chemotherapy Transport to Enhance Tumor Cell Kill
Source: PLoS Comput Biol. 2016 Jun 10;12(6):e1004969. doi: 10.1371/journal.pcbi.1004969 (PMC4902302; doi:10.1371/journal.pcbi.1004969)
Supplement: S1 Text — (DOCX) [file pcbi.1004969.s001.docx]

**Supporting Information**

**SI Text**

**Patient histopathology measurements.** H&E-stained microscopic slides of randomly selected human liver specimens following chemotherapy and resection were obtained from a cohort of 27 patients with colorectal cancer (CRC) metastatic to liver at the MD Anderson Cancer Center (MDACC). Six patients were not included in the final analysis due to the lack of visible dead tumor tissue. Based on the assumption that histologic sections are isotropic, the fraction of tumor killed$f_{\text{kill}}$was directly measured as fraction of the area of dead tumor from the histopathology images along with measurements of radius of blood vessels $r_{\text{b}}$ and BVF for each patient; there were a total of 20 slides for each patient. Measurements were manually performed using GNU Image Manipulation Program (GIMP) [[1](#_ENREF_1)]. An illustration of the measurement of $r_{\text{b}}$ is shown in **Fig. S1A**. In order to calculate the fraction of the area of dead tumor (see **Fig. S1B**), dead tumor regions were colored red, areas of live tumor were colored blue, and the portions that were not tumor (i.e. normal tissue) were colored green, as shown in **Fig. S1C**.

The fraction of dead tumor area $f_{\text{kill}}$was calculated according to [[2](#_ENREF_2)]:

$f_{\text{kill}}$ = # of red pixels / (# of red pixels+ # of blue pixels+ # of green of pixels) (S1)

We compared the $f_{\text{kill}}$ values measured by our pathologist with our computer-based measurements. A cumulative distribution plot was generated to compare the $f_{\text{kill}}$ measurements (**Fig. S2A**). We observed that the pathologist’s $f_{\text{kill}}$ values were shifted to the right to some extent from our measurements. This result is somewhat expected because when pathologists make their measurements of $f_{\text{kill}}$, they normally do not examine whether the normal tissue is destroyed or not, i.e., they only look at the tumor area and determine whether it is dead or live. To correlate with what pathologists would measure in clinical practice, our $f_{\text{kill}}$ values were shifted to the left by 0.108, i.e., the difference between the average of the $f_{\text{kill}}$ measured by us and average of the $f_{\text{kill}}$ measured by the pathologist. Another cumulative distribution plot was generated to compare our new $f_{\text{kill}}$ values with the pathologist’s measurements (**Fig. S2B**). This new, revised set of $f_{\text{kill}}$ values was then used to perform the model fitting to the histopathology data with Mathematica [[3](#_ENREF_3)].

**Pre-treatment contrast CT scans**. Contrast CT scans performed according to standard clinical protocols were acquired *prior to chemotherapy* on 18 patients (from the cohort of 21 CRC patients) at MDACC according to institutional review board (IRB)-approved protocols. The simple average of three Hounsfield Unit (HU) measurements in representative areas within the entire tumor was calculated at each phase of the test for each patient: a late arterial phase (30-35 s after start of contrast injection), a portal venous phase (50-55 s), and a delay phase (minutes, variable timing).

**Calculation of relative error.** An error of 25% was found in the CT measurements; this was estimated by comparing the corresponding measurements from contrast enhancement of aorta (standard deviation in aorta/average of aorta). Thus error bars for the model predictions based on the CT scan data were calculated using this 25% error estimated in CT measurements. The upper and lower limits came from ± 25% of the *predicted* BVFs and this gave rise to an upper and lower limit for $f_{\text{kill}}$, which were then used in the calculation of standard error in *predicted* $f_{\text{kill}}$. The average relative error between the model prediction (P) and the measured kill value (M) was calculated as: $<(f_{\text{kill}}(\text{P})-f_{\text{kill}}(\text{M}))/f_{\text{kill}}(\text{M})>$, and then outliers more than 2 standard deviations from the mean were removed from the calculation.

**Application of the “bolus chemotherapy” model to patient data to estimate model parameters.** In our previous work [[2](#_ENREF_2)], we derived a formula for $f_{\text{kill}}$ based on the simplifying assumption of a drug concentration profile at equilibrium , which solves Eq. 1 (main text) with *φ* = 1 and $\partial\sigma/\partial t\cong0$:

, (S2)

where *K*_0_ and *K*_1_ are modified Bessel functions of the second kind of orders 0 and 1, respectively; all parameters, including, , BVF, and (diffusion penetration length), can be directly measured from histopathology or imaging. This formula, while an approximation, only requires parameter values from one time point after the system equilibrates.

The condition is similar to immediate drug kill, characterized by a large *λ*_k_. If we take a limiting case for $\lambda_{\text{k}}\to+\infty$ and $t\to0$, Eq. 2 (main text) further reduces to , where . Note, however, that the steady-state assumption for Eq. 1 (main text) also demands a fast time scale, which outweighs the requirement of a fast drug killing. That is, the previous model [[2](#_ENREF_2)] additionally requires $\lambda_{\text{u}}<<\lambda_{\text{k}}$, and hence, represents a special case of the generalized model Eqs. 1 and 2 (main text). The limiting conditions toward the model are . Practically, it may also represent a situation where there exists an external time scale much longer than the time scales considered in the model. For instance, if a patient is examined every month, the examination interval is longer than the time scale of drug-induced cell death (presumably in days), and apparently is much longer than the time scale of drug uptake by cells (presumably in minutes).

**References**

1. The GIMP Team (2013) GNU Image Manipulation Program. <http://docs.gimp.org/odftest/en.pdf>.

2. Pascal J, Bearer EL, Wang Z, Koay EJ, Curley SA, et al. (2013) Mechanistic patient-specific predictive correlation of tumor drug response with microenvironment and perfusion measurements. Proc Natl Acad Sci U S A 110: 14266-14271.

3. Wolfram Research (2008) Mathematica, Version 8.0, Mathematics and Algorithms. <http://www.wolfram.com/learningcenter/tutorialcollection/MathematicsAndAlgorithms/MathematicsAndAlgorithms.pdf>.
