# Supplementary figures and images for: Theory and Experimental Validation of a Spatio-temporal Model of Chemotherapy Transport to Enhance Tumor Cell Kill
Source: PLoS Comput Biol. 2016 Jun 10;12(6):e1004969. doi: 10.1371/journal.pcbi.1004969 (PMC4902302; doi:10.1371/journal.pcbi.1004969)

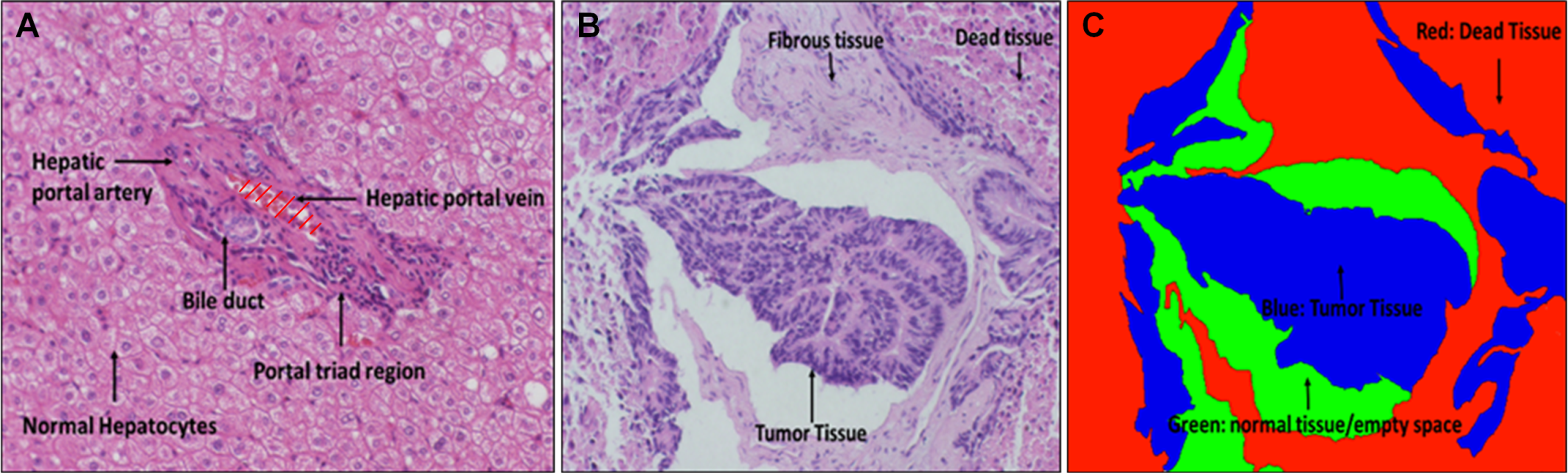

Supplement: S1 Fig — (A) A portal triad in normal liver. (B) Example of a histologic section from one patient. (C) Segmentation of the histologic section B for calculation of the fraction of dead tumor area: dead tumor (red); live tumor (blue); no tumor (green). (TIF) [file pcbi.1004969.s002.tif]

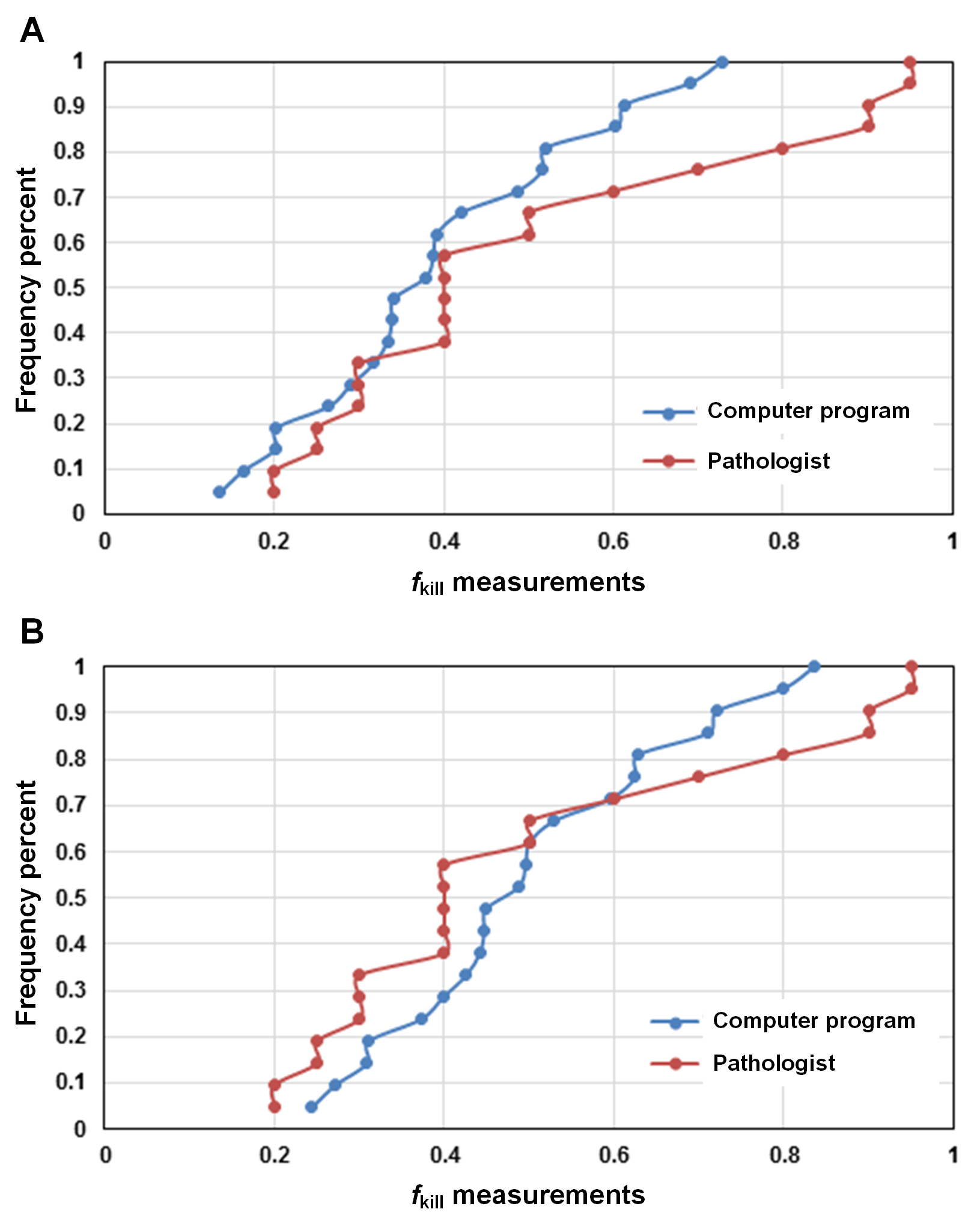

Supplement: S2 Fig — Data were obtained from histopathology images of 21 patients with CRC metastatic to liver. Measurements by image segmentation using the GIMP software (blue); standard clinical assessment by the pathologist at MDACC (red). (A) Measurements of fkill by software vs. by pathologist. (B) Measurements of fkill by software and shifted to the right by 0.108 vs. by pathologist. (TIF) [file pcbi.1004969.s003.tif]

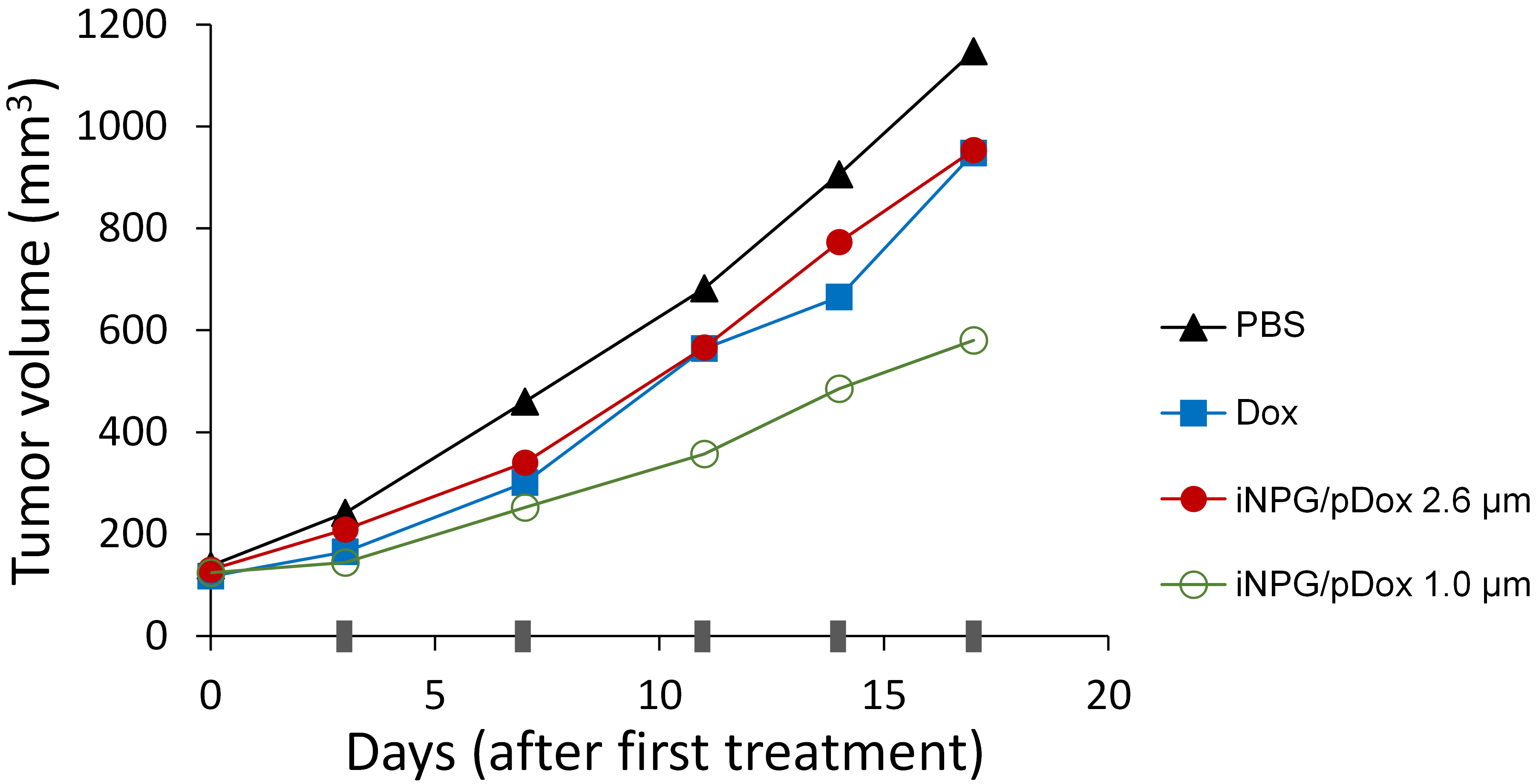

Supplement: S3 Fig — Four treatment groups: PBS (control), free doxorubicin, 1.0 μm porous silicon particle loaded with chemotherapy drug (iNPG/pDox 1.0), and 2.6 μm porous silicon particle loaded with chemotherapy drug (iNPG/pDox 2.6). Data were measured on days 0, 3, 7, 11, 14, and 17 after first treatment. (TIF) [file pcbi.1004969.s004.tif]
